# Variational Pansharpening for Hyperspectral Imagery Constrained by Spectral Shape and Gram–Schmidt Transformation †

**DOI:** 10.3390/s18124330

**Published:** 2018-12-07

**Authors:** Zehua Huang, Qi Chen, Qihao Chen, Xiuguo Liu

**Affiliations:** Faculty of Information Engineering, China University of Geoscience (Wuhan), Wuhan 430074, China; huang_rs@cug.edu.cn (Z.H.); chenqi@cug.edu.cn (Q.C.); chenqihao@cug.edu.cn (Q.C.)

**Keywords:** hyperspectral image, data fusion, variational pansharpening, spectral fidelity, correlation fidelity

## Abstract

Image pansharpening can generate a high-resolution hyperspectral (HS) image by combining a high-resolution panchromatic image and a HS image. In this paper, we propose a variational pansharpening method for HS imagery constrained by spectral shape and Gram–Schmidt (GS) transformation. The main novelties of the proposed method are the additional spectral and correlation fidelity terms. First, we design the spectral fidelity term, which utilizes the spectral shape feature of the neighboring pixels with a new weight distribution strategy to reduce spectral distortion caused by the change in spatial resolution. Second, we consider that the correlation fidelity term uses the result of GS adaptive (GSA) to constrain the correlation, thereby preventing the low correlation between the pansharpened image and the reference image. Then, the pansharpening is formulized as the minimization of a new energy function, whose solution is the pansharpened image. In comparative trials, the proposed method outperforms GSA, guided filter principal component analysis, modulation transfer function, smoothing filter-based intensity modulation, the classic and the band-decoupled variational methods. Compared with the classic variation pansharpening, our method decreases the spectral angle from 3.9795 to 3.2789, decreases the root-mean-square error from 309.6987 to 228.6753, and also increases the correlation coefficient from 0.9040 to 0.9367.

## 1. Introduction

Hyperspectral (HS) images provide abundant and fine spectral information, which can be widely used in many applications, including forest mapping [1], urban environment monitoring [2], ecophysiology assessment [3], and quality control of agriculture [4]. However, the optical remote sensing systems are limited by the amount of incident energy, onboard storage, and bandwidth transmission [5,6]. The spectral and spatial resolution of remote sensing images cannot increase at the same time. Therefore, the HS images generally have lower spatial resolution than other remote sensing images. For this reason, the HS image pansharpening technique is developed to generate a high-resolution HS image by combining a panchromatic (PAN) image and a HS image from the given area.

In HS image pansharpening, to protect the spectral information in a HS image and to further generate the accurate fine spectrums at higher spatial resolution are important problems. If the spectral information of the HS images is distorted in pansharpening, various applications of the pansharpened images are directly affected, including lithological mapping [7], coral reef inversion [8], and discrimination of forest types [9]. The accuracy of interpretation and quantitative inversion based on the HS images will be significantly reduced. Therefore, a preferred HS pansharpening algorithm should be implemented to enhance the spatial detail information in HS images, to protect the spectral information in HS images, and to generate accurate spectral information in high resolution.

Numerous HS pansharpening methods have been proposed over recent decades. On the basis of the underlying principles, current pansharpening methods can be broadly categorized into component substitution (CS), multi-resolution analysis (MRA), matrix factorization, and Bayesian approaches [10,11]. CS approaches separate the spatial and spectral information of the HS images by different transformations. The component with spatial details is replaced by the PAN image, and the components are inversely transformed to reconstruct the pansharpened image. Typical CS approaches include Gram–Schmidt adaptive (GSA) [12], guided filter principal component analysis (GFPCA) [13], etc. These algorithms provide good spatial information [12]. However, due to the wavelength ranges of PAN and HS images are different, radiometric distortion is increased [10]. MRA extract the spatial details by taking the difference between the original and low-pass filtered PAN image. The obtained spatial details are used to enhance the HS image. MRA involves many methods, such as modulation transfer function generalized Laplacian pyramid (MTF_GLP) [14], smoothing filter-based intensity modulation (SFIM) [15]. These methods perform well in robustness and efficiency [16,17], but suffer from aliasing effect and spatial details distortion [18]. Matrix factorization decomposes the images based on the linear spectral mixture model. Then, the endmember matrix of the HS image and the abundance matrix of the multispectral image are multiplied to recover the pansharpened image. Matrix factorization provides good spectral qualities, but only applied in the fusion of multispectral and HS data [19], which is different from pansharpening. Bayesian approaches first define appropriate prior distributions of the images, and obtain the posterior distribution following Bayes’ theorem. Then, the Bayesian estimators are computed for fusing the images. Well-known Bayesian approaches include wavelet-based Bayesian fusion [20], maximum a posteriori (MAP) estimation fusion [21], and variational approach [22]. These methods do not limit the number of bands [23], but suffer from high computational cost and large spectral distortion. 

Variational approach is one of the promising pansharpening methods. The first variational approach is based on three assumptions: the first one describes that the HS and PAN images contain same geometry, the second one represents the relation of the PAN image to each HS bands, and the third one denotes that the low-resolution pixels equal to the convolution and downsampling of high-resolution pixels [22]. Based on the assumptions, the constraint and regularize terms are proposed to construct an energy function, whose optimal solution is the pansharpened image. Following the idea, Moeller et al. [24] extended the variational pansharpening to HS images by building a spectral correlation preserving term based on the definition of spectral angle mapper. Zhang et al. [23] improved the HS variational method by assuming that fused bands should retain the correlation with the upsampled multi-spectral bands. Subsequently, Duran et al. proposed a band-decoupled variational method (NLVD) [25], which uses a constraint to preserve the radiometric ratio between each spectral bands and the PAN image. On the other hand, to effectively preserve spatial information, Chen et al. [26] introduced a dynamic gradient sparsity penalty, which exploits sharp boundaries from PAN images. Liu et al. [27] proposed an optimizing method by integrating spatial fractional-order geometry and spectral–spatial low-rank priors. As a result, this method strengthens both the geometric and spectral preserving constraints. The aforementioned methods can achieve promising results. However, the spectral distortion may be caused by two reasons: (1) the spectral information may be changed in pansharpening process; (2) the spectral information of pansharpened images is much finer than that of the original HS images because of an increase in spatial resolution. Thus, the spectral preservation of variational methods should consider both the spectral feature preserving and the change in spatial resolution. Meanwhile, upsampled HS images often have blocky or blurry artifacts, which may lead to a low correlation between pansharpened and the actual observed images.

The present paper is extended from our previous work [28], designing a variational pansharpening method for HS imagery constrained by spectral shape feature and image correlation. This work focuses on the spectral distortion caused by spatial resolution enhancement and the low correlation between the pansharpened and the actual observed images. Specifically, the two main contributions of this paper are:

- We assume that the change in spatial resolution may lead to spectral information differences between pansharpened and low-resolution HS images. Based on this assumption, the new spectral fidelity term firstly uses the spectral shape feature to characterize the spectral information in the HS image. Then, the pixels in the neighboring window are assigned with different weights. The weights and the spectral shape features of the neighboring pixels are integrated to generate the spectrum at a high spatial resolution.
- A new correlation fidelity term based on GSA method is designed to maintain the correlation between the actual observed high spatial resolution image and the pansharpened image. The result of GSA is used as our correlation constraint, which is obtained by inversely transforming the transformed bands of the HS image and the simulated intensity image.

Afterward, the proposed fidelity terms are combined with the spatial fidelity term in the classic variational pansharpening to construct a new energy function. The optimal solution of this function is the pansharpened image, which is computed by gradient descent method.

The remaining parts of this paper are organized as follows. We briefly introduce the classic variational pansharpening method in Section 2. Our assumptions and the novel variational method is described in Section 3. The evaluation method and the experimental results are presented in Section 4, together with the comparison among the advanced pansharpening methods. The conclusions are provided in Section 5.

## 2. Classic Variational Framework

The classic variational pansharpening framework [22] is briefly introduced in this section. Let $\Omega \subset R^{2}$ be an open bounded image domain. We refer to the PAN image as P: Ω→R. $H=\left(H_{1},H_{2},\ldots H_{i}\right)$ represents the original HS image, and $u=\left(u_{1},u_{2},\ldots u_{i}\right)$ represents the pansharpened image, where *i* represents the *i*th band of the images. *n* denotes the number of the bands of the HS image. On the basis of the definition of image pansharpening, three hypotheses are proposed to describe the relation between the test images and the pansharpened images. Then, the pansharpening problem can be regularized by optimizing an energy function based on the hypotheses.

The first hypothesis states that the geometry of the bands in pansharpened images is contained in the topographic map of corresponding PAN images [25]. Since the pansharpened images and the PAN images contain the same area, the surface objects and features in the two images are nearly the same. Therefore, the geometry information, such as textures and edges, in the PAN images should be in consistent with that in the pansharpened images. The geometry information can be represented by the topographic map, which is acquired by the vector field. Then, the vector field of the PAN image should equal to that of each band in the pansharpened image. The spatial fidelity term is built as follows:

(1) $$E_{g}=\overset{n}{\underset{i=1}{\sum }}\int_{\Omega }\;\left(\left|\nabla u_{i}\right|-\theta \cdot \nabla u_{i}\right),$$

where ∇u is the gradient of the pansharpened image *u*, θ is the normal vector field of the PAN image.

The second assumption expresses that the PAN image is a linear combination of the bands in the HS image with mixing coefficients $\alpha_{1},\ldots ,\alpha_{n}$ [29]. Due to the bandwidth of the PAN image is wider than that of the HS image, the PAN image can be treated as a weighted sum of the narrow bands in the HS image. The weight of each band in the HS image is distributed according to the spectral response of the corresponding channel. On the basis of this idea, the PAN image can be synthesized from the bands in the pansharpened image. Then, the image relation preserving term is derived as follows:

(2) $$E_{r}=\int_{\Omega }\;{\left(\overset{n}{\underset{i=1}{\sum }}\alpha_{i}u_{i}-P\right)}^{2}.$$

The third assumption considers that a low-resolution pixel can be formed from the high-resolution one by low-pass filtering followed by subsampling [25]. Compared with the high spatial resolution image with the same area, the low spatial resolution image consists of less pixels, while the area of each pixel is larger. The low-pass filter makes the pixels contain neighboring information. The subsampling helps reduce the number of the pixels in an image. In this way, the low spatial resolution HS image can be simulated from the pansharpened image. This relation is expressed as follows:

(3) $$E_{f}=\overset{n}{\underset{i=1}{\sum }}\int_{\Omega }\;\Pi_{s}{\left(k_{i}*u_{i}-H_{i}\right)}^{2}dx,$$

where $k_{i}$ is convolution kernel of the low-pass filter, and $\Pi_{s}$ is a down-sampling process using grid *S*. 

Then, the three terms are used to construct the energy function as follows:

(4) $$\begin{array}{cc} E=\sum_{i=1}^{n}\gamma_{n}\int_{\Omega }\;\left(\left|\nabla u_{i}\right|+div\theta \cdot u_{i}\right) & +\lambda \int_{\Omega }\;{\left(\sum_{i=1}^{n}\alpha_{i}u_{i}-P\right)}^{2}\\ & +\mu \int_{\Omega }\;\Pi {\left(k_{i}*u_{i}-H_{i}\right)}^{2}, \end{array}$$

where $\gamma_{i},\lambda ,\mu$ are the weighting coefficients of the corresponding terms. The Equation (4) constraints the properties of the pansharpened image to be consistent with that of the PAN and HS images. Thus, the low energy of this function indicates that the pansharpened image satisfies the desired properties. Many methods have been used to find the minimization and obtain the resulting image, such as gradient descent method and a parabolic equation.

## 3. Proposed Variational Pansharpening

In this section, we first present our assumptions as well as the proposed spectral and correlation fidelity terms. Then, these two terms are used to construct the new energy function for image pansharpening. Subsequently, the numerical method of the energy function is provided.

### 3.1. Spectral Fidelity Term

**Figure 1:**
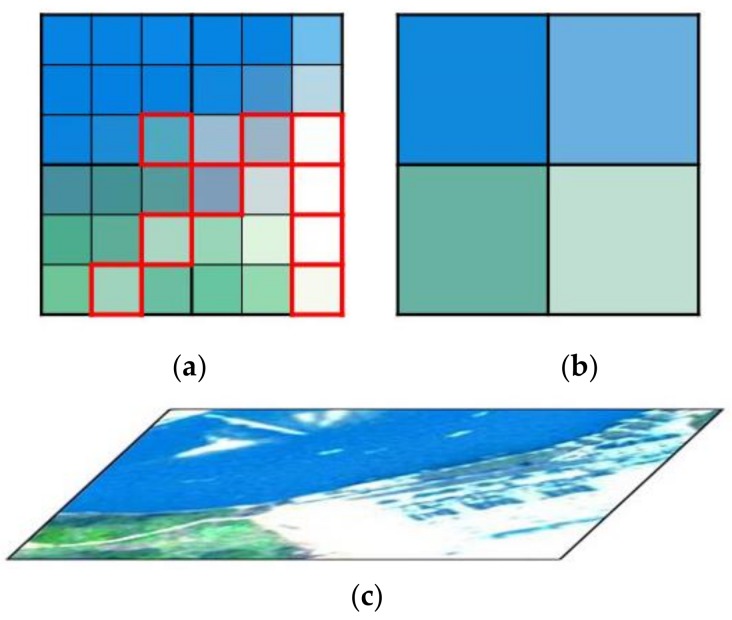
Example of images with different resolutions in the same area: (**a**) high-spatial-resolution image, (**b**) low-spatial-resolution image, and (**c**) the real scene.

Previous works [23,24,30] assumed that the spectral information contained in the pansharpened image should be also contained in the HS image. Hence, the spectral features of the HS image are extracted and directly integrated to the pansharpened images. However, the pansharpened image is required to represent detailed spatial features with fine spectral information because the pansharpened image has much higher spatial resolution than the original HS image. Therefore, we assume that the spatial resolution change may lead to spectral information difference between pansharpened images and low-resolution HS images. As shown in Figure 1a, the spectral features of fine pixels in the red boxes are different from the corresponding low-resolution pixels in Figure 1b. This difference may result from two possibilities: (1) coarse pixels in the original HS images are decomposed into fine pixels in the pansharpened images. The fine pixels at the boundary of a coarse pixel are easily affected by the neighbors in the HS image. (2) If a non-pure pixel is divided, then the spectral information of pure decomposed pixels may be similar to that of the neighboring pixels from the same class rather than that of the original non-pure coarse pixels. Thus, spectral information preservation should also consider spatial resolution change in image pansharpening. In our method, we combine the neighboring pixels in spectral preservation. We first extract the spectral features of the neighboring pixels. Then, the similarity degree between the target pixel and neighboring pixels are measured for distributing weights to the corresponding spectral features. The pixels with higher similarity has larger weights, while the pixels with lower similarity are on the contrary. After that, we use the spectral features of each neighboring pixels to constraint the target pixel and the weights are utilized to define the importance degree of the constraints. The detailed description is provided as follows:

To quantitatively describe the spectral feature, we design the spectral shape feature, which computes the difference between the target band and the mean of all bands in the image. The mathematical expression is as follows:

(5) $$D_{Hi}\left(x\right)=H_{i}\left(x\right)-mean\left(H\left(x\right)\right),$$

where $D_{Hi}\left(x\right)$ is the spectral shape value of the *i*th band of pixel x in image *H*, and $mean\left(H\left(x\right)\right)=\overset{n}{\underset{i=1}{\sum }}H_{i}\left(x\right)/n$ is the mean of all channels. 

The spectral shape feature of the HS image is assumed to be linearly approximate to those of the pansharpened image because pansharpened images retain most of the spectral features of HS images:

(6) $$D_{u}\left(x\right)=\Phi \left(x\right)\cdot D_{H}\left(x\right),$$

where $D_{H}\left(x\right)$ is the spectral shape feature of pixel x in *H*, $D_{u}\left(x\right)$ is the spectral shape feature of the corresponding pixel x in *u*, and Φ(x)=mean(u(x))/mean(H(x)), which is used to match the spectral shape feature of the pixel x in the original HS data with those of the corresponding pixel in the pansharpened data.

**Figure 2:**
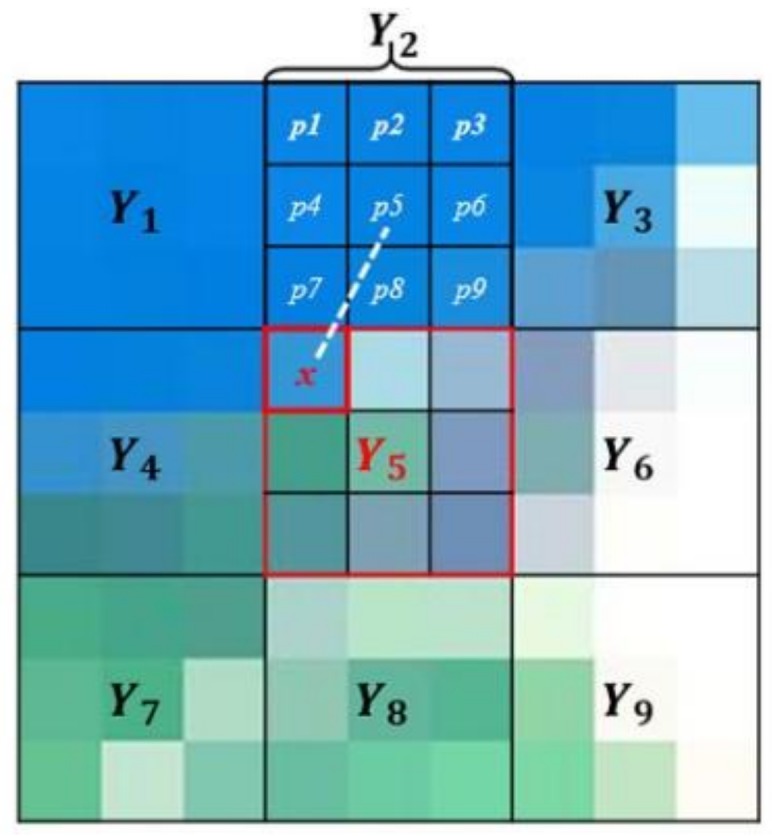
Weight distribution of the coarse pixel $Y_{2}$ in the neighboring window of the target pixel x. Each pixel $Y_{1}\ldots Y_{9}$ in the neighborhood is weighted.

Following the proposed assumption, our method also considers the neighboring pixels to correct the spectral distortion between different spatial resolutions. We use the weights of the neighboring coarse pixels to reflect their influence on the target fine pixel. In previous studies, many weight distribution methods for neighboring pixels have been proposed, such as the Gaussian filtering method [31], the nonlocal denoising method [32], the bilateral denoising method [33], etc. To reflect the spectral similarity, we design a weight distribution method by measuring the distance and the similarity factors between the target pixel and adjacent block of fine pixels. For example, *x* is the target fine pixel required correction, and $Y_{1}\ldots Y_{9}$ are coarse pixels in the neighborhood of *x* (Figure 2). To assess the influence of the coarse pixel $Y_{2}$, we match the location of the coarse pixels to the PAN image. The distance factor measures the Euclidean distance from the target pixel *x* to the center of the coarse pixel $Y_{2}$, which is represented by the white dotted line. The similarity factor calculates the absolute value of the differences between the target pixel *x* and each fine pixel p1,…,p9 covered by $Y_{2}$. The sum of these absolute differences is utilized to represent the spectral similarity of pixels *x* and $Y_{2}$. On the basis of these two factors, we distribute the weight to $Y_{2}$ as follows:

(7) $$w\left(x,Y\right)=\frac{1}{C\left(x\right)}{\left(\int_{Y}\;d_{\rho }\left(x,Y\right)\left|P\left(x\right)-P\left(p\right)\right|dp\right)}^{-1},$$

where *x* is the target fine pixel, *Y* is the neighboring coarse pixel in the neighboring window I, $d_{\rho }\left(x,Y\right)=\sqrt{{\left(A_{x}-A_{Y}\right)}^{2}+{\left(B_{x}-B_{Y}\right)}^{2}}$ represents the Euclidean distance between the pixels, $A_{x}$ and $B_{x}$ are the horizontal and vertical coordinates of pixel *x*, $A_{Y}$ and $B_{Y}$ are the horizontal and vertical coordinates of the center of the pixel Y, the *p* is the fine pixel covered by *Y*, $C\left(x\right)=\int_{I}{\left(\int_{Y}d_{\rho }\left(x,Y\right)\left|P\left(x\right)-P\left(p\right)\right|dp\right)}^{-1}dY$ is a normalizing factor, and *u*(*x*) represents the PAN image value of the pixel *x*. Following this strategy, each coarse pixel $Y_{1}\ldots Y_{9}$ in the neighborhood of *x* is weighted. Pixels with shorter distance and higher similarity have larger weights.

In terms of the spectral shape feature relationship represented by Equation (6) and the weights of the neighboring pixels, we propose the new spectral fidelity term used to generate the accurate spectral information. The differences between the spectral shape feature of the target pixel and that of the coarse HS pixels in the neighborhood are firstly calculate. These differences can be the representations of the spectral errors in the target pixel. Then, we multiply the square of the differences by their corresponding weights to integrate each neighboring pixel’s importance degree. The neighboring coarse pixel with higher similarity will be more concerned in the correction. The spectral information of the pixels could be corrected via minimizing the weighted sum of the squares of these differences. The mathematical expression is the following:

(8) $$E_{s}=\int_{\Omega }\;\int_{I}\;{\left(D_{u}\left(x\right)-\Phi \cdot D_{H}\left(Y\right)\right)}^{2}w\left(x,Y\right)dxdY.$$

In image pansharpening, a small value of this spectral fidelity term indicates a high spectral quality of the fused image.

### 3.2. Correlation Fidelity Term

In previous variational methods, it is assumed that the original HS image could be formed from the ideal pansharpened image by filtering and subsampling. This assumption is often used as the constraint to preserve the correlation between the observed high spatial resolution image and the pansharpened image [6,26]. However, because of the low spatial resolution, both spatial and spectral features in the original HS image are blurred and decimated, which are different from those of the observed high spatial resolution image. The original HS image is not highly correlated with the actual observed high spatial resolution images, especially for the heterogeneous areas. Using the original HS image as correlation constraint could not further improve the quality of the pansharpened images. Therefore, we need to generate a high quality reference to constraint our pansharpened image.

To obtain the high correlation reference image, we fuse the test images previously. The initial fused result is used to build the new correlation fidelity term. Current pansharpening methods are discussed in detail in [5]. The Gram–Schmidt adaptive method [12] performs better than many other methods, such as guided filter principal component analysis, modulation transfer function with generalized laplacian pyramid, etc. Meanwhile, GSA method is fast and easy to be realized. Related methods that have been also mentioned in [5] are Bayesian naive, Bayesian sparse and HySure. Both of them achieve good performances in HS image superresolution. However, these methods are developed for solving the fusion problem of multispectral and HS images, which is different from image pansharpening. The underlying principles of Bayesian and HySure methods are not suitable for integrating a HS image with a single band PAN image. Thus, the GSA method is selected in our pansharpening method to preprocess the test images.

GSA method firstly resamples the HS image to the same size as the PAN image. An intensity image O is calculated by linearly combining different channels of the HS image with weighting coefficients $g_{1},g_{2},\ldots ,g_{n}$. The linear model is:

(9) $$O=\overset{n}{\underset{i=1}{\sum }}g_{i}H_{i}.$$

The coefficients $\left\{g_{i}\right\}$ are estimated using a linear regression between the channels of the HS image and a low-pass filtered PAN image $\tilde{P}$. The liner regression function is

(10) $$\tilde{P}=g_{1}H_{1}+g_{2}H_{2}+\ldots +g_{n}H_{n}+b.$$

Then, we perform the GS transformation on the intensity image O and the HS image. The spatial information of the HS image can be enhanced by substituting the PAN image for the first component of the transform result based on the underlying principle. However, the spectral distortion is proportional to the correlation between the replaced component and the image [12]. Thus, the PAN image is usually matched to the first transform component by histogram-matching before the replacement. After that, we can achieve a new set of components, which consist of the processed PAN image and the GS transform components except the first one. The pansharpened image can be generated by inversely transforming the new set of components.

We construct the new correlation fidelity term by using the GSA result to constraining the pansharpened image. It has to be mentioned that the GSA result is not seen as an ideal high spatial resolution HS image. Spectral distortions and radiation errors are still contained in this result. Nevertheless, comparing with the low resolution HS image, the GSA result could provide a comparatively higher correlation reference, which avoids the influence of the blur and distortion effect in the HS image. The spectral and spatial errors will be corrected by other fidelity terms to further improve the image quality. The new correlation fidelity term is constructed as follows:

(11) $$E_{c}=\int_{\Omega }\;{\left(u-Z\right)}^{2},$$

where Z denotes the previously pansharpened image resulted from GSA. This term enables us to limit the distortion errors globally.

### 3.3. New Energy Function and Numerical Method

We construct a new energy function by combining the novel spectral fidelity term, the novel correlation fidelity term, and the spatial fidelity term in the classic variational pansharpening. The mathematical representation is expressed as follows:

(12) $$E=E_{s}+E_{c}+E_{g}.$$

We extend the expression (4) proposed by Ballester et al. [22] as:

(13) $$E=\gamma \overset{n}{\underset{i=1}{\sum }}\left(\alpha \int_{\Omega }\left|\nabla u_{i}\right|dx+\beta \int_{\Omega }\;div\theta \cdot u_{i}dx\right)+\frac{\eta }{2}\int_{\Omega }\;\int_{I}\;{\left(D_{u}\left(x\right)-\Phi \cdot D_{H}\left(Y\right)\right)}^{2}w\left(x,Y\right)dxdY+\frac{\mu }{2}\int_{\Omega }{\left(u-Z\right)}^{2},$$

where α and β are positive constants that help to adjust the value of the spatial fidelity term [34], *η* and μ are the weighting coefficients of the spectral and correlation fidelity terms, respectively. The function in Equation (13) constraints the difference in the desired properties of pansharpened and ideal images. Thus, the optimal solution of this function is the pansharpened image.

Various methods can be used to search the minimization of the energy function. In our approach, we select the extensively used gradient descent method. Given an initial pansharpened image $u^{0}$, this method iteratively approaches the local minimum value of the energy function by moving in the opposite direction of the gradient. The high-quality pansharpening image is obtained when the gradient descent method reaches the minimization. We firstly compute the first-order differential form of Equation (13) as follows:

(14) $$\frac{\partial E}{\partial u_{i}}=-\gamma \left[\alpha \cdot div\left(\frac{\nabla u_{i}}{{\left|\nabla u_{i}\right|}_{\varepsilon }}\right)-\beta \cdot div\left(\theta \right)\right]+\eta \int_{I}\left(D_{u}\left(x\right)-\Phi \cdot D_{H}\left(Y\right)\right)w\left(x,Y\right)dY+\mu \left(u-Z\right).$$

The locally steepest descent direction is

(15) $$\frac{\partial u_{i}}{\partial t}=-\frac{\partial E}{\partial u_{i}}=\gamma \cdot \alpha \cdot div\left(\frac{\nabla u_{i}}{{\left|\nabla u_{i}\right|}_{\varepsilon }}\right)-\gamma \cdot \beta \cdot div\left(\theta \right)-\eta \int_{I}\left(D_{u}\left(x\right)-\Phi \cdot D_{H}\left(Y\right)\right)w\left(x,Y\right)dY-\mu \left(u-Z\right),$$

where ∆t is the iterative search step. By applying the search step, we obtain:

(16) $$\frac{\partial u_{i}}{\partial t}=\frac{u_{i}^{k+1}-u_{i}^{k}}{∆t}.$$

Finally, we can use the following function to iteratively compute the solution:

(17) $$u_{i}^{k+1}=u_{i}^{k}+∆t\cdot \gamma \left[\alpha \cdot div\left(\frac{\nabla u_{i}}{{\left|\nabla u_{i}\right|}_{\varepsilon }}\right)-\beta \cdot div\left(\theta \right)\right]-∆t\cdot \eta \int_{I}\left(D_{u}\left(x\right)-\Phi \cdot D_{H}\left(Y\right)\right)w\left(x,Y\right)dY-∆t\cdot \mu \left(u_{i}^{k}-Z\right),$$

where $u^{k}$ is the result of the *k*th iteration, and $\Phi^{k}=mean\left(u^{k}\right)/mean\left(H\right)$ is the coefficient of the *k*th iteration. The initial image $u^{0}$ of this iteration scheme is set as the oversampled HS image. As the iteration times *k* increases, $u^{k}$ continuously approaches the high-quality pansharpened image.

### 3.4. Procedures

We present the pseudocode of the proposed method in Algorithm 1, and summarize the workflow as follows:

(1) The original HS image is resampled to the size of the PAN image. The resampled HS and PAN images are input into the proposed method as the test images *H* and *P*, respectively. Then, we compute the spectral shape feature of the *H*.
(2) We compute the gradient field of the PAN image and each channel of the pansharpened image $u^{k}$. The spatial fidelity term is constructed to transfer the geometric information to the pansharpened image.
(3) We compute the spectral shape feature of the pansharpened image $u^{k}$ following Equation (5), and then compute the weights of the neighboring pixels and the coefficient $\Phi^{k}$. The spectral fidelity term is subsequently built to correct the spectral information in $u^{k+1}$.
(4) The test images *H* and *P* are pansharpened previously by GSA as the correlation constraint. The correlation fidelity term is built to preserve the image correlation of $u^{k+1}$.
(5) The modified image $u^{k+1}$ is generated by the iterative process, which consists of the spatial, spectral, and correlation fidelity terms. Subsequently, $u^{k+1}$ is returned to step (2) to reconstruct a new iteration. The process continues until the image u approaches a stable result.

**Algorithm 1** The proposed method1: **Input**: the PAN image *P* and the HS image *H* oversampled to the size of *P*.2: **Initialize**:the pansharpened image $u^{0}\;=\;H$. Fixed ∆*t*, *γ*, *η* and *μ*3: Compute $D_{H}$ following Equation (5).4: Obtain *Z* by GSA5: **for**
***k*** = **1**, **2**, **…**, **N**6:  **for**
***i*** = **1**, **2**, **…**, **n**7:   **for**
**pixel**
*x* ∈ **Ω**8:     Compute *D_u_* following Equation (5).9:    **for**
**pixel**
**y** ∈ ***I***10:     Compute the weights w(x, y) following Equation (7).11:    **end for**12:    Compute the iterative scheme in Equation (17)13:   **end for**14:  **end for**15: **end for**16: ***u*** = ***u*^*k* + 1^**.17: **OUTPUT**: pansharpened image u.

## 4. Experiments and Discussion

In this section, we introduce three test datasets and the evaluation method. Then, the experimental results of three test datasets are presented. The proposed variational pansharpening is compared with the original variational method, NLVD [25] and four other methods in terms of spatial and spectral domains.

### 4.1. Dataset

Experiments are performed on three HS datasets obtained using the HJ-1A and EO-1 satellites. The data of HJ-1A is downloaded from China Centre for Resources Satellite Data and Application [35]. The EO-1 satellite data is provided by United States Geological Survey (Reston, VA, USA) [36]. Each dataset contains a simulated high-resolution PAN image a simulated low-resolution HS image. The first dataset is of Wupin, a county in Fujian Province, with mountains covered with vegetation and dense buildings. The second dataset is of Hong Kong, where the main area is urban and marine. The third dataset is of Wuhan City, in Hubei Province, China, with lakes and an urban area. Table 1 provides a detailed description of test datasets. With these datasets, the performance of our methods in natural, urban, and complex terrains can be evaluated comprehensively.

Several channels of the HS image in the second dataset contain some noise because the Hyperion sensor on the EO-1 satellite has malfunctioned. We manually remove these noisy bands and use the 154 remaining clear bands to develop a new HS image.

### 4.2. Evaluation Indexes

The widely accepted evaluation protocol of image pansharpening is adopted in our research [5]. First, the real HS image is used as the reference image to assess the quality. Then, the test PAN image and the test HS image are simulated. The test PAN image is obtained by spectrally downsampling the reference HS image. The test HS image is obtained by applying a Gaussian blur and downsampling to the reference HS image. Afterward, the test images are fused via different pansharpening methods. The pansharpened images are compared with the reference image to evaluate the spatial and spectral qualities.

We conducted visual and quantitative assessments to evaluate the quality of the resulting image. The visual evaluation can provide a general idea of the results, but can also be easily influenced by individual preference. The quantitative evaluation is objective and accurate, which includes five widely used indexes [5]:

1. *Spectral angle mapper.* SAM criterion calculates the vector angle between the reference and pansharpened images to assess the spectral quality. The ideal SAM is equal to 0. The equation is
  $$\mathrm{SAM}\left(a,b\right)=\arccos\left(\frac{\langle a,b\rangle }{\left||a||\;||b|\right|}\right),$$
  where a and b are vectors.
2. *RMSE*. The RMSE describes the radiometric distortion between the reference and the resulting image. The ideal RMSE is equal to 0. The equation is
  $$\mathrm{RMSE}\left(A,B\right)=\frac{{\left||A-B|\right|}_{F}}{\sqrt{n}},$$
  where ${\left||A|\right|}_{F}$ is Frobenius norm of *A*, *n* is the number of the pixels in *A*.
3. *Relative dimensionless global error in synthesis (ERGAS)*. ERGAS combines root-mean-square error (RMSE) of each channel in images to give a global spectral quality assessment. The ideal ERGAS is equal to 0 [37]. The equation is
  $$\mathrm{ERGAS}\left(A,B\right)=100d\sqrt{\frac{1}{m}\overset{m}{\underset{k=1}{\sum }}{\left(\frac{RMSE}{\mu_{k}}\right)}^{2}}$$
  where *d* is the ratio between the resolutions of the PAN and HS images, *m* is the number of bands, μ is the mean of the band.
4. *Correlation coefficient*. CC evaluates the spatial correlative degree of the reference and the fused images. The ideal CC is equal to 1. The equation is
  $$\mathrm{CC}\left(A,B\right)=\frac{1}{m}\overset{m}{\underset{k=1}{\sum }}\frac{\sum_{j=1}^{n}\left(A_{j}-\mu_{A}\right)\left(B_{j}-\mu_{B}\right)}{\sqrt{\sum_{j=1}^{n}{\left(A_{j}-\mu_{A}\right)}^{2}\sum_{j=1}^{n}{\left(B_{j}-\mu_{B}\right)}^{2}}}$$
5. *Universal image quality index*. The UIQI synthetically measures the loss of correlation, the luminance distortion, and the contrast distortion. The ideal UIQI is equal to 1 [30]. The equation is
  $$\mathrm{UIQI}\left(A,B\right)=\frac{4\sigma_{AB}\bar{A}\bar{B}}{\left(\sigma_{A}^{2}+\sigma_{B}^{2}\right)\left({\bar{A}}^{2}+{\bar{B}}^{2}\right)}$$
  where $\sigma_{AB}$ is the covariance of *A* and *B*, $\bar{A}$ is the mean of *A*.

### 4.3. Analysis of Parameters

The key parameters of our method include the iteration times *k*, the step size *∆t*, and the weighting coefficients *γ*, *η*, *μ*. We firstly conduct a sensitivity analysis of the parameters based on the experiments of dataset 1. The parameters determined in the analysis are expanded to other datasets after fine-tuning. Then, a parameter selection strategy is provided based on the analysis.

#### 4.3.1. Sensitivity of Parameters

**Figure 3:**
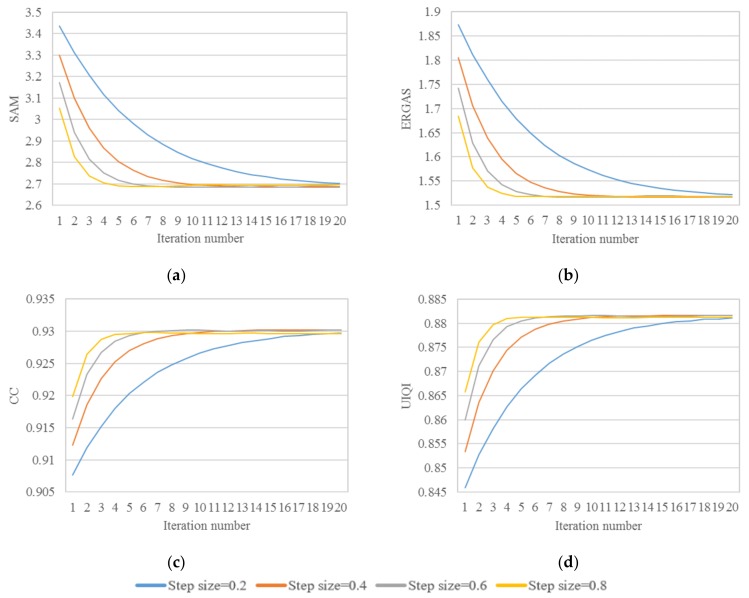
The evaluation indexes vary with the step size and iteration number: (**a**) Spectral angle mapper (SAM), (**b**) ERGAS, (**c**) Correlation coefficient (CC), (**d**) Universal image quality index (UIQI).

Parameter *k* determines the iteration times; with the increase of *k*, the pansharpened image gradually approaches the ideal result. Parameter *∆t* is used to control the size of each iteration. A small step size could correct small distortions to further improve the result quality. To find the optimal step size and iteration numbers, we temporarily set the weighting coefficients to fixed values. The experimental results are shown in Figure 3, when the step size is larger than 0.6, the evaluation indexes oscillate within a small range after 10 iterations. This is because the large step size could not meet the accuracy requirement when the pansharpened image is close to the boundary. On the contrary, a small step size necessitates many iterations to gradually correct the errors, which will significantly increase the amount of calculations. In Figure 3, it can be seen that when the step size is set to 0.6, the SAM and ERGAS continuously decrease, and the CC continuously increases. Comprehensively considering the computational amount and the result quality, we set k=10 and ∆t=0.6.

After the step size and iteration times were determined, we discussed the weighting coefficients. γ, η and μ are utilized to combine the spatial fidelity, the spectral fidelity and the correlation fidelity suitably. The influence of each weighting coefficient are explained as follows: if γ increases, the image is corrected with enhanced spatial information in the pansharpening process; when η increases, the importance of the spectral constraint is strengthened; as μ increases, the correlation degree is cumulatively preserved. The reason for this outcome is that the correction values of the fidelity terms are increased by large weight in the subsequent iterative correction. To further discuss the sensitivity of the weighting coefficients, we provided experiments when one of the coefficients is equal to zero. These special cases are compared with the general case that γ=0.3, η=0.4, μ=0.3. The quantitative evaluations are presented in Table 2. The bold font represents the general case. When γ=0.3, the spatial constraint is weakened, the spatial quality of the pansharpened images are reduced. The CC and UIQI decrease to 0.9104 and 0.8647, respectively. Meanwhile, due to the weighting coefficient of the spectral fidelity term increases, the result achieves better spectral quality. The SAM is improved from 2.6843 to 2.5752. When η=0, the spectral fidelity term could not effectively correct the spectral information in the pansharpened image, which lead to large spectral distortion. In Table 2, the SAM increases to 3.0327, the ERGAS and RMSE increase to 1.5279 and 278.1249, respectively. The spatial quality constrained by the spatial term basically remains the same. When μ=0, the pansharpened images are not constrained by the correlation fidelity term. The CC and UIQI are 0.7790 and 0.6561, respectively, which are both decreased. The ERGAS and RMSE also achieve worse performances. Thus, the high correlation constrain is important in combining the spectral and spatial fidelity terms to produce pansharpened images with higher comprehensive quality.

The weighting coefficients γ, η and μ are set by fine-tuning. The experiments show that the most reasonable weighting coefficients are γ=0.3, η=0.4 and μ=0.3, which achieves a satisfied overall quality of the resulting image.

According to above analysis, the step size was set to 0.6, the iteration number was set to 10, and the weighting coefficients were set to γ=0.3, η=0.4 and μ=0.3. Then, we expanded the parameters to dataset 2 and dataset 3. We can achieve ideal pansharpened images by spending a little time tuning the parameters. The parameters of the experiments are determined after fine tuning. In our approach, the iteration number, the three weighting coefficients, and the step size of dataset 2 are 10, 0.4, 0.4, 0.2 and 0.6, respectively. In dataset 3, these parameters are 10, 0.3, 0.35, 0.35, 0.6, respectively. Because ideal results can be achieved by fine-tuning the parameters, overfitting did not occur in our experiments.

#### 4.3.2. Selection of Parameters

Based on the above analysis, we present a selection strategy of parameters for the proposed method. Due to the complexity and diversity of different images, it is difficult to design a general function to obtain the accurate optimal parameters. Meanwhile, the weighting coefficients are used to balance the spatial and spectral quality of the results according to the actual requirements. Therefore, the parameter selection strategy provides the ranges of the optimal parameters. The exact values of the parameters are obtained by tuning manually in these ranges.

The weight coefficient of the correlation fidelity term could be decided based on the correlation between the PAN image and the HS image. If the mean CC between each band of the HS image and PAN image is relatively high, more weights can be distributed to the spatial fidelity term to enhance the spatial details. When the mean CC is low, the weight coefficient of the correlation fidelity term should be larger to improve the image correlation. The weight of the spectral fidelity term is usually defined between 0.3 and 0.4, which is a little larger than the others. The reason is that the spectral quality is important in image pansharpening. Additionally, to keep the balance the spectral and spatial information, the weights of each term should be in the range from 0.2 to 0.4. Then, we can provide a parameter selection strategy in Table 3.

### 4.4. Results and Comparison

The novel variational pansharpening method is compared with advanced methods including GFPCA [13], SFIM [15], GSA [12], MTF_GLP [14], the classic variational pansharpening [22] and NLVD [25]. The first five compared methods are implemented by a downloadable MATLAB toolbox [38]. The NLVD is implemented by programing in MATLAB. 

**Figure 4:**
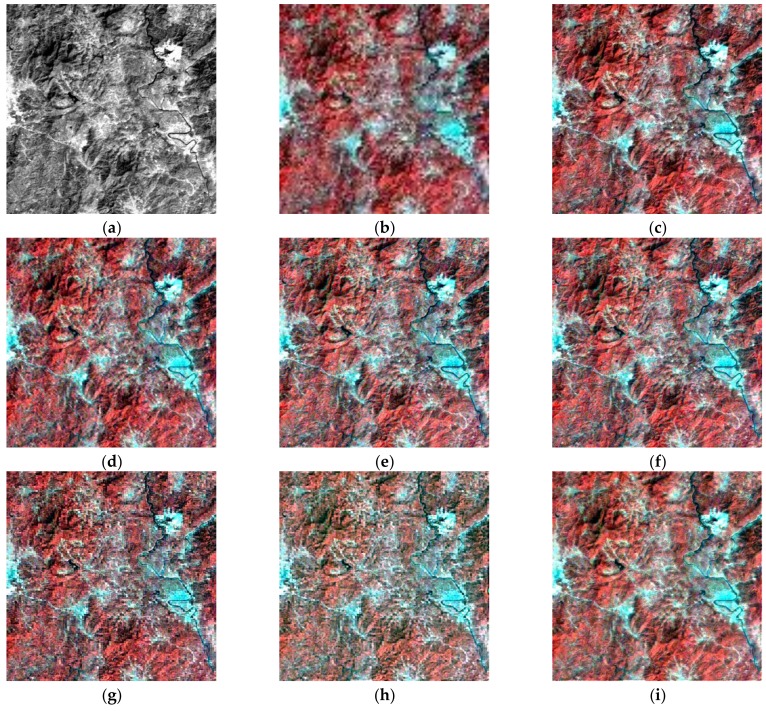
Test images and the pansharpened images of dataset 1: (**a**) test PAN image (0.5–0.9 μm), (**b**) test HS image (0.504–0.929 μm), (**c**) proposed method, (**d**) classic variational method, (**e**) guided filter principal component analysis (GFPCA), (**f**) Gram–Schmidt adaptive (GSA), (**g**) smoothing filter-based intensity modulation (SFIM), (**h**) modulation transfer function generalized Laplacian pyramid (MTF-GLP), (**i**) band-decoupled variational method (NLVD).

**Figure 5:**
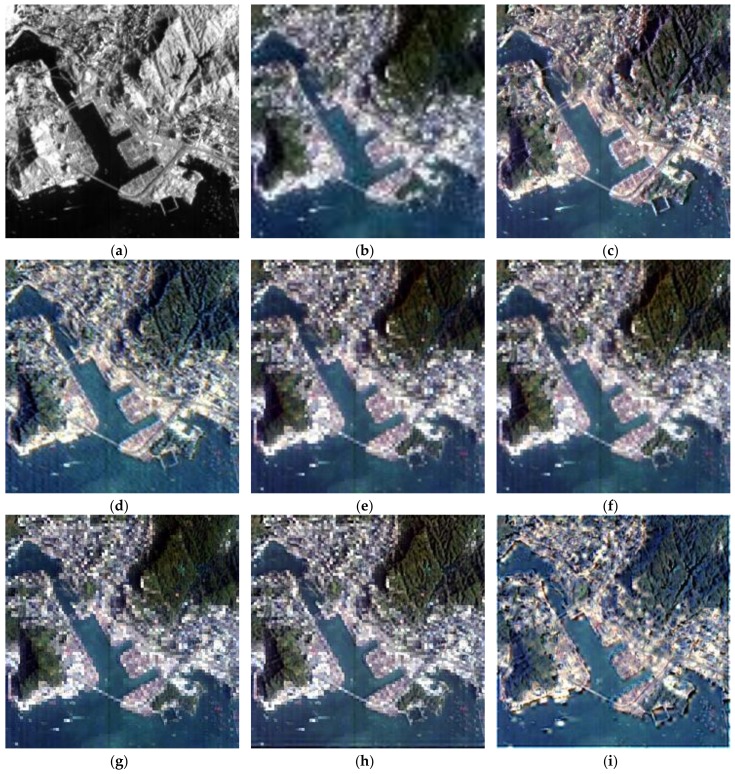
Test images and the pansharpened images of dataset 2: (**a**) test PAN image (0.5–0.9 μm), (**b**) test HS image (0.446–2.342 μm), (**c**) proposed method, (**d**) classic variational method, (**e**) GFPCA, (**f**) GSA, (**g**) SFIM, (**h**) MTF-GLP, (**i**) NLVD.

**Figure 6:**
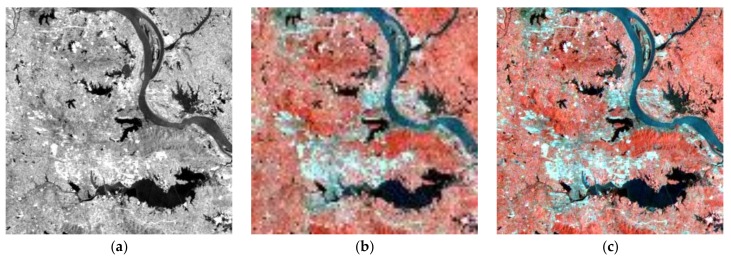

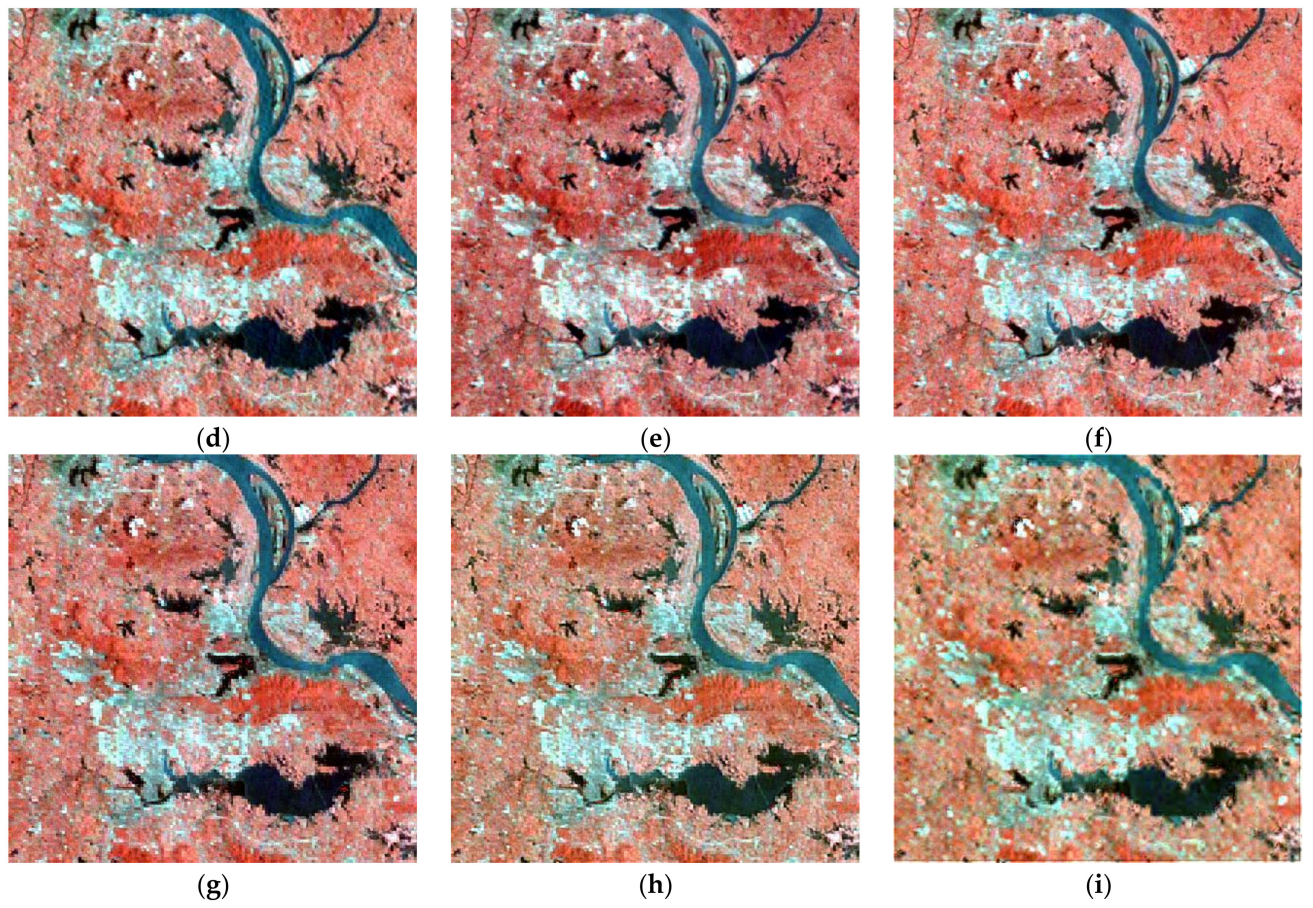
Test images and the pansharpened images of dataset 3: (**a**) test PAN image (0.5–0.9 μm), (**b**) test HS image (0.504–0.929 μm), (**c**) proposed method, (**d**) classic variational method, (**e**) GFPCA, (**f**) GSA, (**g**) SFIM, (**h**) MTF-GLP, (**i**) NLVD.

**Figure 7:**
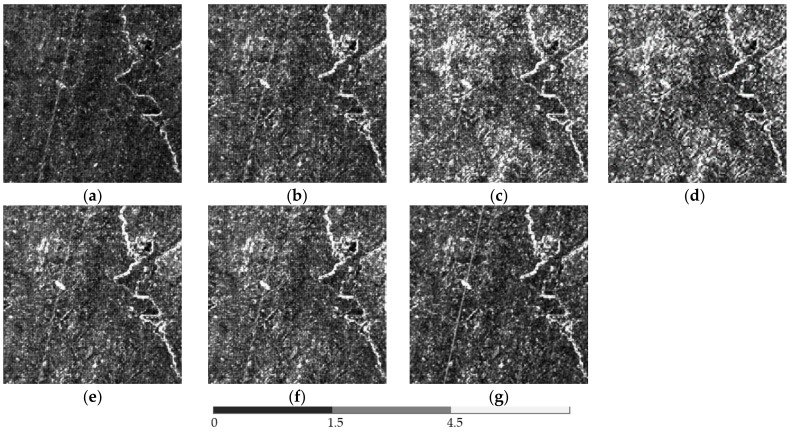
SAM distribution image of dataset 1: (**a**) proposed method, (**b**) classic variational method, (**c**) GFPCA, (**d**) GSA, (**e**) SFIM, (**f**) MTF-GLP, (**g**) NLVD.

**Figure 8:**
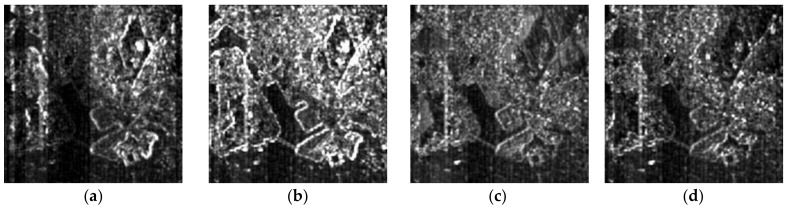

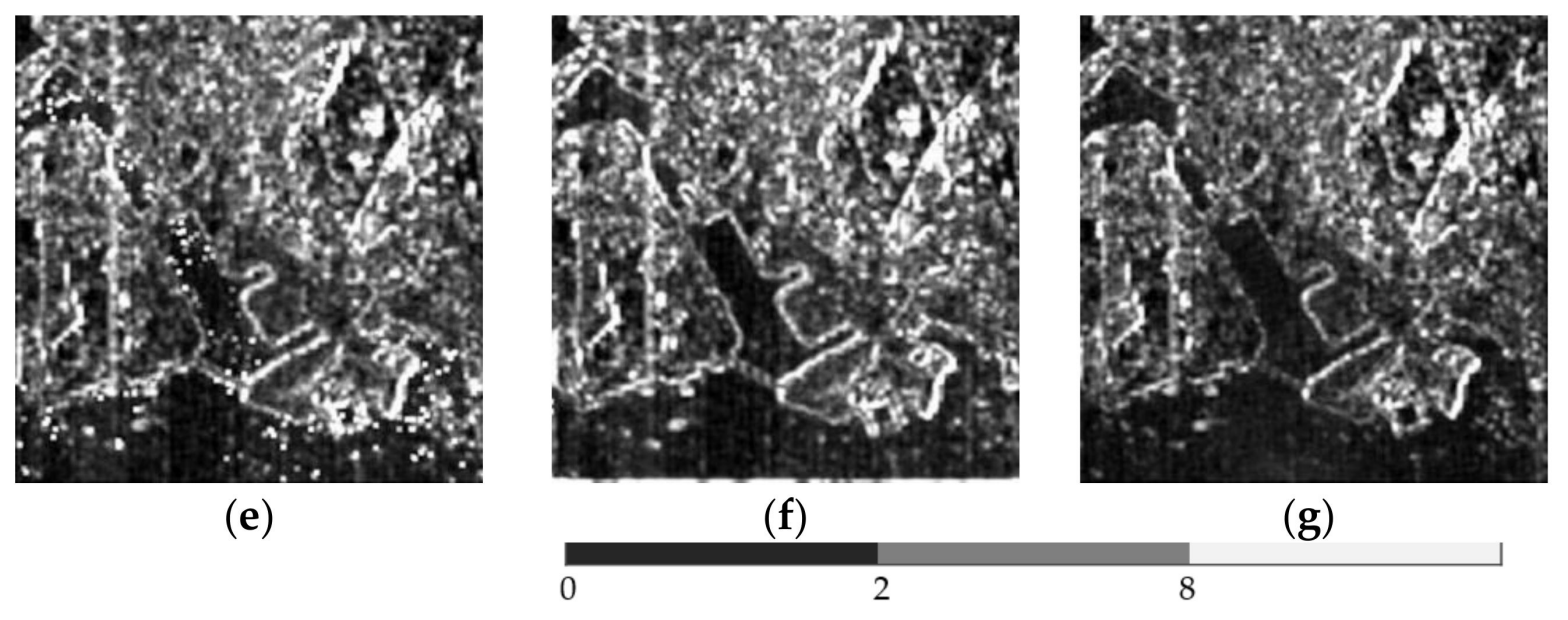
SAM distribution image of dataset 2: (**a**) proposed method, (**b**) classic variational method, (**c**) GFPCA, (**d**) GSA, (**e**) SFIM, (**f**) MTF-GLP, (**g**) NLVD.

**Figure 9:**
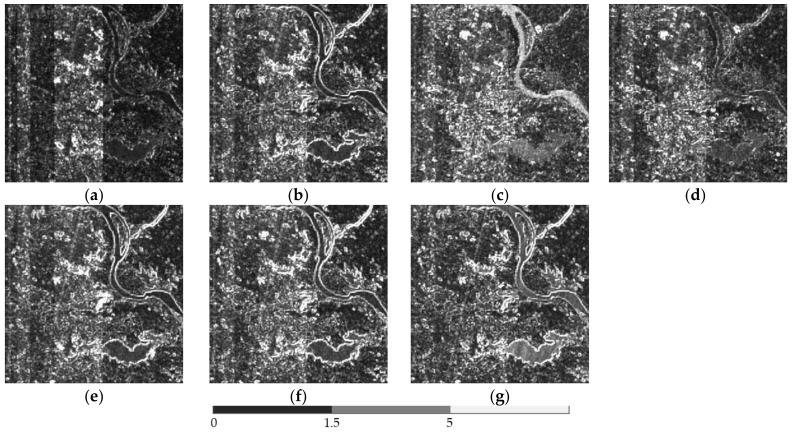
SAM distribution image of dataset 3: (**a**) proposed method, (**b**) classic variational method, (**c**) GFPCA, (**d**) GSA, (**e**) SFIM, (**f**) MTF-GLP, (**g**) NLVD.

**Figure 10:**
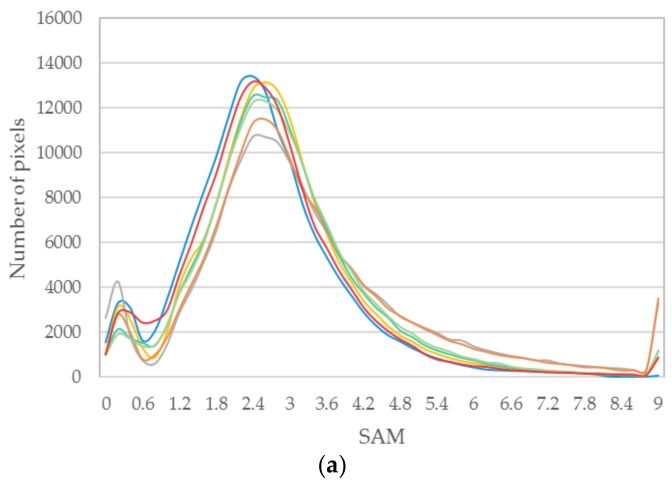

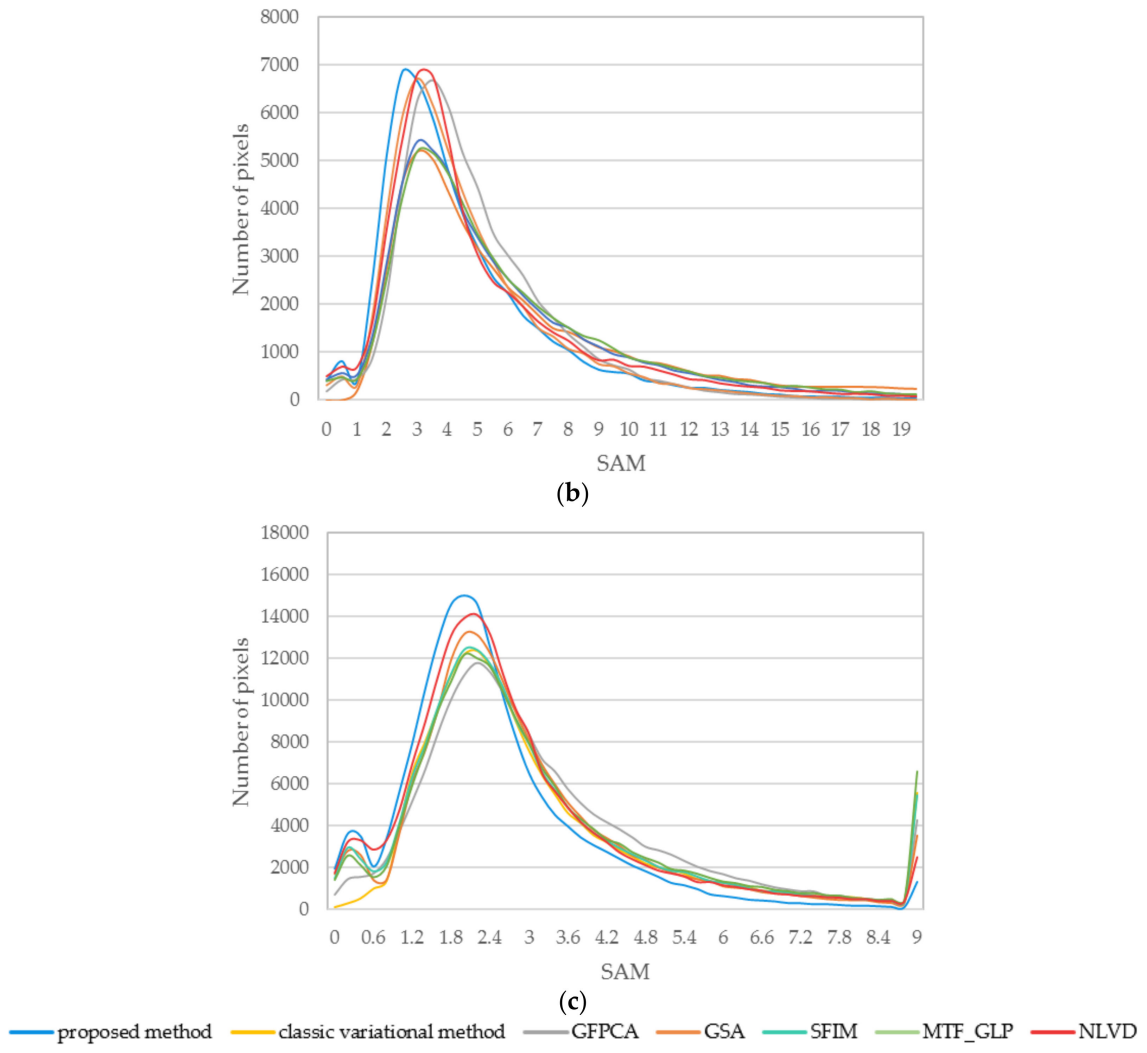
Histograms of SAM: (**a**) dataset 1, (**b**) dataset 2, (**c**) dataset 3.

The above six methods are tested on each dataset in the experiments. Color images are composited from the results of the three datasets (Figure 4, Figure 5 and Figure 6, respectively). Table 4, Table 5 and Table 6 provide the quantitative performances. The best method is denoted by bold font in the tables. Table 7 presents the means and standard deviations (SDs) of the evaluation indexes of the three datasets, and the highlighted numbers represent the best method. Figure 7, Figure 8 and Figure 9 illustrate the SAM distribution of the three datasets. Figure 10 shows the histogram of SAM. We compare the test methods in terms of their spatial and spectral performance.

#### 4.4.1. Spatial Performance

In visual evaluation, in comparison with the original HS images, the quality of all spatial information in the pansharpened images is improved significantly. GFPCA and GSA provide clear river edges and detailed mountain texture in Figure 4e,f, and all of the features have noticeable boundaries. SFIM preserves the color information of the HS image, but the excessive smoothing phenomenon makes the visual effect blurry. Many ground objects, particularly the buildings in the urban area in Figure 5g, are difficult to be identified. By contrast, MTF_GLP retains a large number of details, but suffers from severe color distortion. For example, the color of the mountain in Figure 6h is much different from that of the tested HS image. The classic variational method reduces color distortion but degrades visual effects, especially along the edges of rivers and lakes (Figure 6d). The spatial details generated by NLVD are blurrier than those of GSA and PCA, but better than SFIM and MTF_GLP (Figure 4i). The proposed variational method has clear spatial information comparable with that of GFPCA and GSA. The color of the mountains in Figure 4c and the spatial structures in Figure 5c are well preserved. In addition, the performance of the spatial details, such as mountain area and river in Figure 6, is similar to that in Figure 4. Therefore, the proposed method, GFPCA, and GSA yield the best performance in terms of visual comparison.

In the quantitative evaluation, CC and UIQI represent the spatial quality of the pansharpened images. GFPCA and GSA show good visual results comparable with those of our method, but the CCs of these two methods are 0.9111 and 0.9329 (Table 7), respectively, which are worse than the CC (0.9367) achieved by our method. The CC and UIQI of SFIM are 0.7927 and 0.7161, respectively, which are the worst (Table 7), because of the smooth and blurring effects. MTF_GLP generates color distortion and blocky effects. In accordance with this result, the UIQI of MTF_GLP is 0.7567 (Table 7), our method is superior to this method. The CC of NLVD is 0.8885 (Table 7), which are worse than GFPCA and GSA. Thus, the spatial performance of the proposed variational method is significantly better than that of the other methods. It is noted that the CC and UIQI of our method in Table 5 are a little worse than those of GSA. The reason is that the dataset 2 covers an urban area with complex artificial buildings and diverse fine objects, which result in abundant spatial details and various spectral features. In the pansharpened images, the enhancement of the spatial details usually leads to the spectral distortion. To preserve complex spectral information in the HS image, we strength the spectral fidelity term and weaken the correlation fidelity term by adjusting the weighting coefficients. Thus, the spatial information of the proposed is comparable to GSA, and the spectral quality of our method is better than GSA. In Table 7, the CC and UIQI of our method are 0.9367 and 0.8781, respectively, which are the best among the six methods.

Since the results of GSA are used to construct the correlation fidelity term in our method, the results of GSA are also compared with the low spatial resolution HS images. Table 8 presents the means of the evaluation indexes of the results of GSA and the low spatial resolution HS images on three datasets. The mean CC of the GSA is 0.9326, which is much higher than 0.8284 of the low resolution HS image. Meanwhile, the other indexes SAM, ERGAS, RMSE and UIQI of GSA are both better than those of the low spatial resolution HS image. These quantitative results demonstrate that the results of GSA have higher correlation and smaller errors than the low resolution HS images. Thus, by using the result of GSA as the correlation constraint, the global quality of the pansharpened images can be further improved.

In comparison with those of the classic variational methods, the mean CC of the proposed method increases from 0.9040 to 0.9367, and UIQI increases from 0.8127 to 0.8781. Table 7 shows the dispersion statistics. The SDs of the CC and UIQI in our approach are 0.0172 and 0.0293, respectively, which are close to the results of most of the methods. These results demonstrate that the novel pansharpening method outperforms the other methods in both visual and quantitative evaluation.

#### 4.4.2. Spectral Performance

For better visualization, we present the SAM error image in Figure 7, Figure 8 and Figure 9. Bright pixels represent large SAM errors. The number of bright pixels in the error images of GFPCA and GSA is more than that of the other methods (Figure 7 and Figure 9), showing the largest spectral distortion among the pansharpening methods. SFIM and MTF_GLP produce large error pixels on the edge of the river and the city (Figure 7 and Figure 9), thereby reducing the overall spectral quality of the images. The classic variational method has less bright pixels in the SAM distribution images, but is also more than our method. The images of NLVD are very close to the variational method (Figure 7 and Figure 9). The proposed variational pansharpening method obtains the darkest error image, which indicates the best result. Figure 10 illustrates the histograms of SAM and shows that the SAMs of most of the pixels in our method are 2.4, 2.5, and 2, respectively (Figure 10a–c), which are less than that of the other methods. We also significantly reduce the number of the pixels whose SAM is larger than 8.4 (Figure 10a,c). Thus, our method effectively improves the spectral quality of the pansharpened images.

In quantitative evaluation, SAM, ERGAS, and RMSE are used as the measurements of the spectral fidelity degree. SFIM obtains the poorest quantitative performance (Table 7). In MTF-GLP, consecutive levels of the pyramid are utilized to enhance the spatial details of the HS image, but this method results in low spectral accuracy. The SAM and ERGAS of MTF_GLP are 4.1992 and 6.6298, respectively. The performance of GFPCA and GSA is better than that of SFIM and MTF_GLP, and the mean SAMs in GFPCA and GSA are 3.8388 and 3.5774, respectively. The spectral indexes of NLVD have advantages over GFPCA and GSA. The SAM, ERGAS, and RMSE of our method both rank first among the six methods, indicating that our method provides the best spectral quality.

In comparison with the classic variational method, the means (Table 7) demonstrates that the SAM of our method decreases from 3.9795 to 3.2789, the ERGAS of our method decreases from 5.6598 to 4.0983, and the RMSE of our method decreases from 309.6987 to 228.6753. These quantitative results prove that our method noticeably improves the accuracy of the spectral information, and performs better than the other methods. In the dispersion statistic, the SDs of the SAM, ERGAS, and RMSE of our method are 0.8177, 3.3791 and 107.6446, respectively. The SDs of ERGAS and RMSE are the lowest among all of the methods. Thus, the spectral quality of the different datasets generated by our method is more stable than that of the other methods.

#### 4.4.3. Overall Performance

GFPCA and GSA preserve the spatial information well but produce large spectral distortion. The classic variational method and SFIM generate blocky and blurring effects. MTF_GLP produce color distortion. NLVD provided worse spatial qualities than the proposed method. The performance of the novel pansharpening method is better than that of the other methods. We summarize the advantages of our method as follows:

1. The novel method uses a spatial fidelity term to constraint the spatial details of the pansharpened image, which generates clear object edges and textures. The mean values of UIQI and CC are 0.8781 and 0.9367, respectively, which proved that our method noticeably improved the spatial quality.
2. The spectral information is corrected by the new spectral fidelity term, which considers the spectral change caused by spatial resolution difference between the resulting and HS images. In spectral quantitative evaluation, our method achieves the best SAM, ERGAS and RMSE results in the comparison.
3. The proposed method designs a correlation fidelity term to improve the image correlation. This term can be combined with the other terms to achieve a better overall quality. The results prove that our method not only achieves good spectral quality but also obviously improves spatial details.

We implement all the test methods in MATLAB using a desktop computer with a Windows 10 system, a 3.3 GHz Intel Core i5 CPU and 8 GB RAM. The processing time of each method are shown in Table 9. The mean processing time of the classic variational method, GFPCA, GSA, MTF_GLP, SFIM, NLVD and the proposed method are 18.66, 5.76, 3.42, 1.7, 2.99, 26.17 and 16.84 s (Table 9), respectively. The speed of the proposed method ranks the fifth, which is higher than other variational approaches (the classic method and NLVD). The efficiency of the proposed approach should be acceptable for most general applications. The proposed variational pansharpening method can obtain better overall quality than GFPCA, GSA, MTF_GLP, SFIM and NLVD with acceptable consuming time.

### 4.5. Main Features of the Proposed Method

We propose some improvements on our previous conference paper [28] in this study. The two main improvements of the proposed method are the spectral constraint combined with neighboring pixels and the new correlation constraint. Due to the consideration of spatial resolution enhancement, the spectral fidelity term in the proposed method utilizes the spectral information of neighboring pixels and a weight distribution strategy to further decrease the spectral distortion. The method in our previous work [28] directly correct the spectral information according to the HS image, which may lead to large error in edge pixels. The other improvement in this study is designing a new correlation fidelity term. Compared with the fidelity terms in our previous work, the new correlation fidelity term based on GSA could provide images with better quantitative performance.

Many previous pansharpening methods have been adopted for evaluation and comparison. In comparison with other methods, this study focuses on the spectral distortion caused by the spatial resolution enhancement and the low correlation between the pansharpened and high resolution actual observed images. The corresponding fidelity terms are specifically designed for improving the qualities of pansharpened images. Hence, our method achieves both better spatial and spectral qualities than other methods. The other feature of our approach is that the spatial and spectral information is jointly enhanced in the process. In this way, the pansharpened images could achieve a better comprehensive information quality than other methods.

## 5. Conclusions

In this paper, we proposed a novel variational pansharpening method for HS imagery constrained by spectral shape feature and image correlation. First, we propose the constraints of spectral shape feature and the correlation preservation. Second, the spectral and correlation fidelity terms are constructed. Finally, we combine these fidelity terms to build the new energy function, and obtain the resulting image based on the optimal solution. Our main contributions are that the new spectral fidelity term and the new correlation fidelity term are designed in the variational framework. The spectral fidelity term uses the difference between bands and the mean value of images to correct the spectral distortion. We assumed that the spatial resolution change may lead to spectral information difference. The neighboring pixels are also considered by distributing the weights in this term. The correlation fidelity term preserves the correlation of the pansharpened image based on the GSA method. 

Experiments on three datasets from HJ-1A and EO-1 satellites show that the novel method performs well in terms of spatial and spectral fidelity. The overall performance of the proposed method is better than the comparative methods, including GSA, GFPCA, MTFGLP, SFIM, the classic variational pansharpening and NLVD. In the quantitative evaluation, the mean values of SAM, ERGAS, RMSE, CC and UIQI (in Table 7) of the proposed method are 3.2789, 4.0983, 228.6753, 0.9367 and 0.8781, respectively, which have been both improved from 3.9795, 5.6598, 309.6987, 0.9040 and 0.8127 of the classic variation method. The mean computation time of our method are 16.84 s. The algorithm efficiency meets the practical requirements in applications.

However, the performance of the proposed method is limited by two problems. First, the enhancement of the spatial details may lead to the distortion in spectral information. The mutual promotion between the spatial and spectral qualities is difficult to be realized by the current constraints. Second, the proposed spectral fidelity terms may produce large errors when processing mixed pixels. In the future work, we will focus on developing new constraint, which could integrate the spatial and spectral information to improve the comprehensive quality of pansharpened images. In addition, Automatic parameter optimization method will be further considered in our future studies.

## Figures and Tables

**Table 1:** The test datasets.

Dataset ID	Dimensions	Spatial Resolution	Bands	Satellite	Longitude/°E	Latitude/°N
1	PAN 400 × 400	100 m	1	HJ-1A	116.08–116.48	24.88–25.24
	HS 100 × 100	400 m	92	HJ-1A		
2	PAN 240 × 240	30 m	1	EO-1	114.09–114.17	22.31–22.37
	HS 80 × 80	90 m	154	EO-1		
3	PAN 420 × 420	100 m	1	HJ-1A	114.81–115.25	30.03–30.40
	HS 140 × 140	300 m	92	HJ-1A		

**Table 2:** Sensitivity of parameters on dataset 1.

Parameters	SAM	ERGAS	RMSE	CC	UIQI
γ=0, η=0.5, μ=0.5.	2.5752	1.3776	253.3581	0.9104	0.8647
γ=0.5, η=0, μ=0.5.	3.0327	1.5279	278.1249	0.9301	0.8773
γ=0.5, η=0.5, μ=0.	2.3609	3.2364	592.8869	0.7790	0.6561
γ=0.3, η=0.4, μ=0.3.	**2.6843**	**1.5167**	**270.2535**	**0.9302**	**0.8816**

**Table 3:** Selection of parameters.

Dataset	CC	γ	η	μ
1, 3	<0.8	0.2–0.3	0.3–0.4	0.3–0.4
2	>0.8	0.3–0.4	0.3–0.4	0.2–0.3

**Table 4:** Quantitative performance of dataset 1.

Method	SAM	ERGAS	RMSE	CC	UIQI
Proposed method	**2.6843**	**1.5167**	**270.2535**	**0.9302**	**0.8816**
Original method [22]	2.7796	1.7719	324.2705	0.9099	0.8438
GFPCA [13]	3.2890	1.7109	315.2741	0.9086	0.8532
GSA [12]	3.1974	1.6204	295.0699	0.9215	0.8635
SFIM [15]	2.9136	2.7048	484.4754	0.8145	0.7213
MTF_GLP [14]	2.9645	2.6916	482.5487	0.8310	0.7453
NLVD [25]	2.7392	1.6982	289.9658	0.8916	0.8631

**Table 5:** Quantitative performance of dataset 2.

Method	SAM	ERGAS	RMSE	CC	UIQI
proposed method	**4.4352**	**8.8718**	**81.0621**	0.9196	0.8407
original method [22]	6.1116	12.5169	140.4907	0.8618	0.7244
GFPCA [13]	4.9522	10.2929	100.5043	0.9042	0.8232
GSA [12]	4.6117	8.9068	83.5041	**0.9248**	**0.8459**
SFIM [15]	6.0753	772.3017	357.9770	0.7413	0.6702
MTF_GLP [14]	6.3742	13.2112	154.5545	0.8550	0.7374
NLVD [25]	4.5564	9.0819	92.8317	0.8777	0.8255

**Table 6:** Quantitative performance of dataset 3.

Method	SAM	ERGAS	RMSE	CC	UIQI
proposed method	**2.7172**	**1.9063**	**334.7104**	**0.9602**	**0.9121**
original method [22]	3.0473	2.6906	464.3349	0.9403	0.8698
GFPCA [13]	3.2751	3.3076	516.7360	0.9206	0.8648
GSA [12]	2.9230	2.2761	390.4692	0.9525	0.9004
SFIM [15]	3.1468	15.1770	2508.2	0.8223	0.7570
MTF_GLP [14]	3.2588	3.9866	675.5911	0.8698	0.7876
NLVD [25]	2.8763	2.1873	382.8934	0.8962	0.8548

**Table 7:** Statistics of the quantitative performance.

Method	Statistics	SAM	ERGAS	RMSE	CC	UIQI
proposed method	mean	**3.2789**	**4.0983**	**228.6753**	**0.9367**	**0.8781**
	SD	0.8177	3.3791	**107.6446**	0.0172	0.0293
original method [22]	mean	3.9795	5.6598	309.6987	0.9040	0.8127
	SD	1.5115	4.8632	132.6098	0.0323	0.0633
GFPCA [13]	mean	3.8388	5.1038	310.8381	0.9111	0.8470
	SD	0.7873	3.7267	169.9548	**0.0069**	0.0175
GSA [12]	mean	3.5774	4.2678	256.3477	0.9329	0.8699
	SD	**0.7400**	3.4912	128.2743	0.0139	0.0227
SFIM [15]	mean	4.0452	263.3945	1116.8841	0.7927	0.7161
	SD	1.4386	359.8877	985.1634	0.0365	0.0356
MTF_GLP [14]	mean	4.1992	6.6298	437.5648	0.8519	0.7567
	SD	1.5427	4.6837	215.0774	0.0160	0.0220
NLVD [25]	mean	3.3906	4.3225	255.2303	0.8885	0.8478
	SD	0.8262	**3.3713**	120.9376	0.0078	**0.0161**

**Table 8:** Means of evaluation indexes of GSA and low resolution hyperspectral (HS) image.

Data	SAM	ERGAS	RMSE	CC	UIQI
Low resolution HS image	4.0617	7.4759	507.6172	0.8284	0.7417
GSA [12]	3.5574	4.2678	256.3477	0.9329	0.8699

**Table 9:** Computational times of pansharpening methods (second).

Dataset	Proposed Method	Original Method [22]	GFPCA [13]	GSA [12]	SFIM [15]	MTF_GLP [14]	NLVD [25]
1	18.05	19.60	6.31	3.77	1.87	3.26	28.24
2	10.87	12.62	3.91	2.24	1.09	2.21	18.01
3	21.59	23.76	7.06	4.26	2.14	3.51	32.25
Mean	16.84	18.66	5.76	3.42	1.7	2.99	26.17
